# Lead Exposure Survey Tool (LEST): A methodological approach to highlight the invisible lead poisoning

**DOI:** 10.1016/j.mex.2023.102432

**Published:** 2023-10-20

**Authors:** Waleed S. Al Sukaiti, Mohammed Abdullah Al Shuhoumi, Hassan I. Al Balushi, Musa Al Faifi, Ziad Kazzi

**Affiliations:** a Department of Emergency Medicine, Ibri Regional Hospital, MOH, Oman; b Oman Poison Control Center, MOH, Muscat, Oman; c Emory University School of Medicine, Atlanta, GA, USA; d Medical Laboratory Scientist and Research Focal Point, Laboratory Department, Ibri Hospital, Oman; e Academic Lecturer, Oman College of Health Sciences, Oman; f Reviewer and Member at Centre of Studies and Research, Oman; g Department of Emergency Medicine, Sohar Hospital, MOH, Oman; h Aseer Central Hospital KSA, Saudi Arabia; i Georgia Poison Control Center, Atlanta, GA, USA

**Keywords:** Methods, Lead poisoning, MENA, Systematic review and expert's consensus, Scientifically-designed tables for further data analysis such as SPSS and R. It is the ultimate outcome that reached after comprehensive analysis of systematic review and expert consensus

## Abstract

Lead is the most common heavy metal found in the Earth's crust. Lead has been widely dispersed and incorporated in the natural world since prehistoric times. In the majority of wealthy countries, the amount of lead entering the atmosphere has been significantly reduced. Acute lead exposure becomes relatively low, but chronic lead exposure remains a substantial public health hazard. Disadvantaged people, are developing and industrializing countries in the Middle East and North Africa (MENA). Between 1981 and 2018, a comprehensive literature search was undertaken in the PubMed and Scopus databases. All studies were evaluated equally based on predetermined inclusion and exclusion criteria. The Delphi method was used to identify numerous resources of lead pollutants. The Mixed Method Appraisal Tool (MMAT) was used to evaluate the quality of identified papers for inclusion in the systematic review synthesis. The studies and sources of lead toxicology were further evaluated using a scale of evidence to establish the degree of evidence based on SIGN & GRADE standards. There were 14 genres and 82 subgenres identified. Through a comprehensive analysis, our cohort developed an exposure survey tool that takes into account the local sociocultural aspects of MENA countries, which will serve as a resource forresearchers,

medical toxicologists, and public health professionals in the MENA region to enhance early detection of potential subjects, conduct further studies and implement exposure prevention strategies.

• There is no single tool available to detect the invisible lead poison. Lead poisoning is a serious public health problem that can have devastating consequences.
• The tool, called the Lead Exposure Survey Tool (LEST), uses a combination of but not limited to data sources, including blood lead levels, environmental lead levels, and demographic information, to identify subjects who are at risk of lead poisoning. LEST is a powerful tool that can help to improve early detection and prevention of lead poisoning.
• The development of LEST is a major breakthrough in the fight against lead poisoning. This tool has the potential to save lives and improve the health of high risk subjects around the world.

## Graphical abstract

**Figure:**
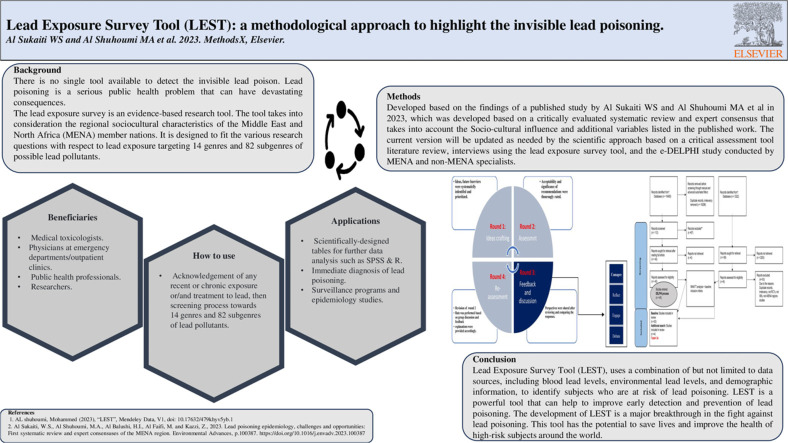

Specifications table

**Table:** 

Subject:	Environmental Science
Specific subject area:	Health, Toxicology, Management, Monitoring, Policy and Law Emergency Medicine, Epidemiology, Public Health and Health Policy.
Name of your method:	Scientifically-designed tables for further data analysis such as SPSS and R. It is the ultimate outcome that reached after comprehensive analysis of systematic review and expert consensus.
Name and reference of original method:	Not applicable.
Resource availability:	Repository name: Mendeley data Data identification number: 10.17632/479khyv5yb.1 Direct URL to data: https://data.mendeley.com/datasets/479khyv5yb/1 [1].

**Method details**

## Why middle east and north Africa countries (MENA) demand a massive attention and standalone analysis?

In the MENA region, cultural norms are unique and distinct from other compartments of the world. The MENA region has its own environment, cross-sectional adjustment, and languages (Arabic more prevalent), and it is a region that is more dominant by Islam and its values and principles.

## What the lead exposure survey tool can be used for?

The lead exposure survey tool can be utilized by researchers, medical toxicologists, and public health experts in the MENA region to conduct lead related research and put exposure preventive measures into motion. Physicians at emergency departments and outpatient clinics could benefit from the survey questions to identify possible exposure to lead that will eventually aid their therapeutic decisions. Researchers can implement the tool that was designed to fit various study designs.

## What is lead exposure survey tool?

The lead exposure survey is an evidence-based research tool. The tool takes into consideration the regional sociocultural characteristics of the middle east and north Africa (MENA) member nations. It is designed to fit the various research questions in respect to lead exposure.

## How was the lead exposure survey tool developed?

A group of scientists from MENA countries launched the lead exposure survey method in 2023 [2]. The current version (2023) was developed based on the findings of a published study by Al Sukaiti WS and Al Shuhoumi MA et al. in 2023, which was developed based on a critically evaluated systematic review and expert consensus that takes into account the Socio-cultural influence and additional variables listed in the published work. The current version will be updated as needed by the scientific approach based on a critical assessment tool literature review, interviews using the lead exposure survey tool, and the e-DELPHI study conducted by MENA and non-MENA specialists.

## What are the requirements?

Because the lead exposure survey tool is generated based on findings of a comprehensive study in the MEAN region that takes several factors only applicable to the MENA countries, it is advised to have the tool utilized in the MENA region only, especially for the research purpose. Clinicians from non-MENA regions could benefits from the tool questionnaire to aid their diagnosis and narrow down the list of suspected diagnoses. Not to mention how essential it would be for clinicians and epidemiologists from MENA and non-MENA region to trace the element of exposure that could take due to a travel in the MENA region given the fact that some lead pollutants are known to be used in the MENA countries only.

## How to use the tool?

This document consisted of two parts:

### I)

- The first part of the tool is considered an initial interview of the patient/subject to obtain demographic characteristics, and acknowledgement of any recent or chronic exposure or/and treatment to lead.
- This part is not considered a formal consent for research subject as every study design required its own informed consent considering the variation in research methodologies and that every subject need to be informed with the study details. A typical informed consent should be formulated according the followed methodological approach and should include the following:
  1. Investigators are advised to use as simple and plain a language and terminology as feasible when writing informed consent letters.
  2. Headers should include “Informed Consent” followed by the title of the study, followed by principal investigator details or institutional review board, purpose of the study, procedures, benefits, risks, compensations if any, confidentiality agreement, contact information, voluntary participation statement, and ultimately the signature of both participant and investigator long with the date of signature.
  3. Page numbers must appear in footers. If your consent letter has more than one page, the footer should include have a place for the participant's initials.
  4. Make sure to include all necessary foundational elements of informed consent that apply to your study design. All components must be added if they pertain to your study.

### II)

- The second part of the tool comprises a screening towards several lead pollutants sources. The answers are shortened to “YES” or “NO”, or “Can't tell”.
- In all “YES” answers, subjects/patients are required to state the time frame or spatial framework to the questions accordingly.

## Limitations

It is not advocated to have the patients/subjects answering the tool by themselves. Due to the reasons:

- Intellectual ignorance in terms of terminologies.
- Clinicians/Researchers are more competent to comprehend the terminologies and for that some questions might not be clear to everyone; hence it is advocated to explain whenever needed.
- In case of minors, rightful guardians are consented to proceed with the survey tool. However, questions are directed to both according to the situation, as guardian might be more competent to explain the question to the minor based in former knowledge of the subject/patient psychology and slang language. Thus, the like hood of obtaining accurate results is more.
- The feeling of being targeted and the feeling of being handled a task that need to be fulfilled as soon as possible, which can trigger answering random answers of YES and NO regardless of the questions being asked.

## Part I

**Table:** 

	Subject charachteristics
1	Patient Name
2	Medical record number
3	Age
4	Gender
5	Nationality

##  

**Part II**

**Table:** 


*(continued on next page)*

**Table:** 

## Ethics statement

The study was conducted in accordance with the Declaration of Helsinki.

## CRediT authorship contribution statement

**Waleed S. Al Sukaiti:** Conceptualization, Resources, Data curation, Formal analysis, Writing – original draft, Writing – review & editing. **Mohammed Abdullah Al Shuhoumi:** Conceptualization, Resources, Data curation, Formal analysis, Writing – original draft, Writing – review & editing. **Hassan I. Al Balushi:** Conceptualization, Resources, Data curation, Formal analysis, Writing – review & editing. **Musa Al Faifi:** Conceptualization, Resources, Data curation, Formal analysis, Writing – review & editing. **Ziad Kazzi:** Conceptualization, Resources, Data curation, Formal analysis, Writing – review & editing.

## Declaration of Competing Interest

The authors declare that they have no known competing financial interests or personal relationships that could have appeared to influence the work reported in this paper.

## Data Availability

Data will be made available on request.
